# Association between Bullying Victimization and Aggression in Lebanese Adolescents: The Indirect Effect of Repetitive Negative Thinking—A Path Analysis Approach and Scales Validation

**DOI:** 10.3390/children10030598

**Published:** 2023-03-21

**Authors:** Feten Fekih-Romdhane, Diana Malaeb, Abir Sarray El Dine, Ecem Yakın, Souheil Hallit, Sahar Obeid

**Affiliations:** 1 Faculty of Medicine of Tunis, Tunis el Manar University, Tunis 1068, Tunisia; 2 The Tunisian Center of Early Intervention in Psychosis, Department of Psychiatry “Ibn Omrane”, Razi Hospital, Manouba 2010, Tunisia; 3 College of Pharmacy, Gulf Medical University, Ajman P.O. Box 4184, United Arab Emirates; 4 Department of Biomedical Sciences, School of Arts and Sciences, Lebanese International University, Beirut P.O. Box 146404, Lebanon; 5 Centre d’Études et de Recherches en Psychopathologie et Psychologie de la Santé, Université de Toulouse-Jean Jaurès, UT2J, 5 allées Antonio Machado, 31058 Toulouse, France; 6 School of Medicine and Medical Sciences, Holy Spirit University of Kaslik, Jounieh P.O. Box 446, Lebanon; 7 Applied Science Research Center, Applied Science Private University, 11931 Amman, Jordan; 8 Research Department, Psychiatric Hospital of the Cross, Jal Eddib P.O. Box 60096, Lebanon; 9 Department of Social and Education Sciences, School of Arts and Sciences, Lebanese American University, Jbeil P.O. Box 13-5053, Lebanon

**Keywords:** bullying victimization, aggression, repetitive negative thinking, validation, students, adolescence

## Abstract

(1) Background: The purpose of the present study was to validate the Perseverative Thinking Questionnaire (PTQ) and the Buss–Perry Aggression Questionnaire-Short Form (BPAQ-SF) and test whether repetitive negative thinking plays an indirect role in the relationship between bullying victimization and aggression among Lebanese adolescents. (2) Methods: This cross-sectional study was conducted between January and May 2022 and included 379 Lebanese adolescent students (64.9% females, mean age 16.07 years). (3) Results: The three-factor solution of the PTQ and the four-factor solution of the BPAQ-SF showed excellent model fit. PTQ mediated the association between bullying victimization and physical aggression, verbal aggression, hostility, and anger. (4) Conclusions: This study expands on previous research by showing that repetitive negative thinking, an impactful socio-cognitive factor for students’ mental health, has a mediating (indirect) effect on the cross-sectional relationship between bullying victimization and aggression. This suggests that interventions aiming to prevent aggressive behaviors among adolescent students may be more effective if focused on repetitive negative thinking.

## 1. Introduction

Bullying victimization is defined as “repeated aggressive behavior, with an imbalance of power between the aggressor and the victim” [1]. It is a specific form of interpersonal violence where the victims are the target of aggression by dominant peers for the intentional purpose of inflicting harm while not being able to defend themselves [2]. Bullying victimization mostly occurs in school, and during early adolescence [3,4,5], and it has several forms. It can be physical (e.g., pushing, hitting, fighting), verbal (e.g., threatening, calling names, spreading rumors, teasing), sexual (e.g., harassment), or social (e.g., exclusion, ignoring) [6]. A high but variable prevalence of this phenomenon has been documented among children and youth worldwide. The marked variability in rates across countries has been explained by several factors, including research design, inconsistencies in definitions, and measurement tools used to assess bullying victimization [7]. 

Multiple studies from many countries have reported different prevalence rates of bullying victimization among adolescents aged 12–18 years: less than 10% in Asian countries (Hongkong, Taiwan, and Macao) [8], 17.9% in Pakistan [9], around 20% in the United States [10], more than 40% in African countries (Malawi and Ghana) [11,12], 40.6% in Philippines [13], 46.9% in Indonesia [14], and 47% in New Zealand [15]. The UNESCO report (2019) estimated that 32% of children and adolescents are experiencing bullying victimization in some form worldwide [16]. A meta-analysis by Modecki et al., encompassing 80 studies that investigated bullying involvement rates of students (aged between 12 and 18 years), revealed wide variation in prevalence rates across contexts, with mean prevalence rates of 36% for victimization [17]. Even though there is still significant under-representation of developing countries of the Arab world in the research literature related to school bullying, similar variations in estimates have been documented, with prevalence rates of 7% in Jordan [18], 11.7% in Tunisia [19], 16% the United Arab Emirates [20], 9.9–20.6% in Algeria [21], and 49.1% in Lebanon [22]. Thus, Lebanon seems to have one of the highest prevalence rates of bullying in the Arab region and even worldwide, with almost 1 in 4 Lebanese adolescents having been estimated to be involved in bullying [23]. Therefore, Lebanon offers particularly interesting social and cultural contexts within which to investigate bullying victimization in adolescent students.

Because of the potential detrimental effects on adolescents’ health [24,25,26], there has been growing and widespread public concern in schools about victimization over the last few years. Indeed, bullying victimization can cause a broad range of consequences, including impaired academic performance [27] and poor general health [25], as well as other internalizing [28] and externalizing [29] mental health problems. Internalizing problems involve depression, anxiety, stress [30,31], self-harm [32], and suicidal ideation and behaviors [33]; while externalizing problems include rule-breaking behaviors and aggression [34,35]. Aggression, specifically, is a harmful and serious consequence that deserves attention from scholars in research to further enhance the knowledge and understanding of its potential influencing factors and possible prevention methods.

### 1.1. Bullying Victimization and Aggression 

Aggression refers to behavior with the purpose of harming another individual [36]. Aggression can take various forms, representing its different aspects: physical and verbal aggression (behavioral and instrumental aspects), anger (affective and emotional aspects), and hostility (cognitive aspect) [37]. The relationship between bullying victimization and aggression has become a subject of growing interest in both the clinical and academic worlds. Prior evidence has shown that victimization and the perpetration of aggression are strongly correlated; and, more importantly, that bullying victimization is a potential risk factor for future perpetration [38]. Previous studies support that bullying victimization, particularly when experienced early in life (in childhood or early adolescence) can lead to aggression over time (e.g., [39,40]). For instance, longitudinal studies have shown that a subset of youth who have been victims of bullying in childhood and early adolescence were more likely to later become perpetrators of bullying, themselves [38,41,42,43]. Various psychosocial factors may contribute to aggressive behavior among victimized adolescents [44]; however, these factors remain largely under-researched and unclear. Elucidating the possible mechanisms underlying the association between victimization and aggressive behavior is important for more than one reason. First, aggression has reached highly alarming rates in schools [45] and has become one of the major problems of today’s society [46,47]. Particularly, previous studies found that Lebanese adolescent students reported moderate to high aggression in 34.0% and 31.9% of cases, respectively; which is higher than rates reported by students from other countries [48]. This is mainly due to the multiple stressors Lebanese youth are facing, including ongoing conflicts, economic crises, and political instability [49]. Second, aggression has been shown to lead to detrimental outcomes in adolescents’ development and mental health [50], increase the likelihood of later offending [51], and have high social and economic costs [52]. Third, the existing intervention programs targeting bullying victimization and aggression have proven to have poor effectiveness in decreasing or preventing these behaviors, especially in adolescents [53,54], suggesting that new psychological interventions need to be developed based on new or under-explored pathways from bullying victimization to aggression. The current study focuses on one possible pathway through which victimization can lead to aggression: repetitive negative thinking. In particular, we hypothesized that bullying victimization could have an indirect effect on aggression via repetitive negative thinking.

### 1.2. Repetitive Negative Thinking as a Mediator between Bullying Victimization and Aggression 

Although only a very limited amount of research is available on the role of socio-cognitive factors in the relationship between bullying victimization and aggression, the little existing evidence suggests that such factors may be determinants for the identification of adolescents at heightened risk of becoming aggressors (e.g., [55,56]). Indeed, victimization has proven to trigger negative emotions and be related to maladaptive coping strategies, both of which are linked to aggression [55]. Additionally, bullying victimization, particularly when repeated over time, is linked to distinct social-cognitive patterns [56]. Maladaptive socio-cognitive processes, such as rumination, self-evaluations, and hostile attribution bias, appear to play major roles in negative outcomes from bullying victimization experiences [57,58,59,60]. 

Repetitive negative thinking is a cognitive process defined as excessive and perseverative thinking about one’s negative experiences or problems (current, past, or future) that are experienced as intrusive and difficult to control [41,61]. It represents an emotion regulation strategy involved in the development and maintenance of several negative mental health problems (e.g., [62,63,64,65]). Previous studies mainly focused on the mediating role of repetitive negative thinking in the relationship between stressors and internalized problems (e.g., [66]). More specifically, repetitive negative thinking has been found to mediate the association between bullying victimization and depression [67]. Studies on externalized problems, however, are more limited. Recently, a prospective longitudinal Finnish study found that more frequent bullying victimization was associated with later bully perpetration through the indirect mediating effect of rumination [68]. Repetitive negative thinking has recently been gaining potential interest within contemporary research related to students’ mental health [69]. We believe that providing empirical support for the hypothesis that repetitive negative thinking plays a mediating role in the relationship between victimization and aggression could offer potentially promising avenues for dealing with aggressive adolescents in school settings.

### 1.3. Bullying Victimization, Aggression, and Repetitive Negative Thinking Assessment Measures 

Some bullying assessment measures have been translated to Arabic and validated in Arab contexts, such as the Revised Olweus Bully/Victim Questionnaire (OBVQ-R) [18,70], the School Climate Bullying Survey (SCBS) [71,72], and the Illinois Bully Scale (IBS) [73]. The Perseverative Thinking Questionnaire (PTQ) [74] and the Buss–Perry Aggression Questionnaire-Short Form (BPAQ-SF) [37] are the most widely used measures of repetitive negative thinking and aggression among adolescents, respectively. The PTQ has been previously validated in languages other than English, including French [75], Spanish [76], Peruvian [69], and Polish [77]. Similarly, different versions of the Buss–Perry Aggression Questionnaire-Short Form (BPAQ-SF) exist, including Portuguese [78], Hungarian [79], Turkish [80], Chinese [81], Spanish [82], and Thai [83]. However, to the best of our knowledge, no research instruments are yet available in Arabic to assess aggression and repetitive negative thinking.

The Arabic language is an official language in 27 states and is spoken by more than 420 million people worldwide [84]. An Arabic version of the PTQ and the BPAQ-SF would allow clinicians to assess these important constructs in different Arabic-speaking contexts, and allow researchers “to compare research findings from different countries and in different languages” [85].

### 1.4. The Present Research 

Adolescent students are in a developmental time period of increased susceptibility to both bullying victimization [86] and aggressive behavior [87]. Therefore, understanding pathways linking bullying victimization and aggression is essential to optimize biopsychosocial development and prevent negative outcomes for this vulnerable population [88,89]. Most of the previous studies in this regard have investigated a unique form of aggression, bullying perpetration, in relation to bullying victimization. However, it has been shown that aggression, in general, is distinct from bullying perpetration, in particular, in terms of definitions and outcomes [90]. We intended, through the present work, to examine bullying victimization in relation to the broad construct of aggression in its multifaceted forms (physical, verbal, anger, hostility), as opposed to one specific form of aggression (i.e., bullying perpetration). Furthermore, given that both bullying [91] and aggression [92] are culturally dependent concepts, cross-cultural differences in the relationship between these two entities might also exist. Hence, it is important to investigate these entities across different cultural backgrounds, particularly under-researched ones. Through the present study, we build on the above-mentioned previous research by exploring whether repetitive negative thinking may be a cognitive factor that plays an indirect role in the relationship between bullying victimization and aggression. Another objective of this study was to validate the PTQ and BPAQ-SF scales among Lebanese Arab-speaking adolescents. We hypothesized higher levels of bullying victimization to be significantly and positively associated with repetitive negative thinking, which in turn was hypothesized to be associated with a higher tendency toward aggression.

## 2. Materials and Methods

### 2.1. Study Design and Participants

This cross-sectional study was conducted between January and May 2022. An online questionnaire was created via google forms software and included information related to the aims of the study as well as instructions for filling out the questionnaire. The initial respondents were then asked to recruit other participants within the same age range (required to participate in the study) and preferably as diverse as possible with regard to their place of habitat within the Lebanese governorates. No credits were offered for participation. The snowball technique was used during sampling. All participants (*N* = 379) were adolescent students (aged between 13 and 17 years old) residing in Lebanon (including all Lebanese governorates: Beirut, Mount Lebanon, North, South, and Bekaa).

### 2.2. Ethical Aspect

The study protocol was approved by The Ethics and Research Committee of the Psychiatric Hospital of the Cross (HPC-035–2020). All respondents were asked to get their parents’ consent and provide electronic informed consent prior to completing the survey. All respondents and their parents were informed about the study’s objectives and general instructions. All procedures were in accordance with the ethical standards of the institutional and/or national research committee as well as with the 1964 Helsinki Declaration and its later amendments or comparable ethical standards. 

### 2.3. Minimal Sample Size Calculation

Using the formula suggested by Fritz and MacKinnon [93] (i.e., $n=\frac{L}{f2}+k+1$, where *f* = 0.26 for a small to moderate effect size, *L* = 7.85 for an α error of 5% and power β = 80%), a minimal sample of 127 was deemed necessary based on 10 variables to be entered in the model.

### 2.4. Study Instruments

The first part of the questionnaire provided information regarding the aims of the current study and the anonymity of collected responses. All participants were required to select the option stating “I got my parents’ approval and consent to participate in this study” to be directed to the questionnaire.

The second part of the questionnaire contained sociodemographic information about the participants (age, gender, governorate, current self-reported weight and height). BMI was calculated according to the World Health Organization formula [94]. The physical activity index is the cross result of the intensity, duration, and frequency of daily activity [95]. The household crowding index, reflecting the socioeconomic status of the family [96], is the ratio of the number of persons living in the house over the number of rooms in it (excluding the kitchen and the bathrooms). To assess financial burden, respondents were asked to answer the following question: “How much pressure do you feel with regard to your personal financial situation in general?” on a scale from 1 to 10, with 10 referring to overwhelming pressure.

The third part included the following questionnaires used in the current study:

**The Illinois Bully scale (IBS).** The IBS, validated in Lebanon [97], is an eighteen-item scale used to assess bullying perpetration (e.g., “I annoyed other students”) and bullying victimization (e.g., “Other students beat and pushed me”) [73]. Questions were scored as follows: “never = 0 and up to seven times or more = 4”. Subscale scores were computed by summing the respective items. Higher scores on these subscales indicated higher bullying perpetration and victimization, respectively [98]. In this study, only the bullying victimization subscale was used (Cronbach’s alpha = 0.91).

**The Buss–Perry Aggression Questionnaire-Short Form (BPAQ-SF).** The BPAQ-SF [99] is a short version of the BPAQ, and it contains 12 items rated on a 5-point Likert scale. The items are organized into four subscales assessing physical aggression (3 items; e.g., “I have threatened people I know”), verbal aggression (3 items; e.g., “My friends say that I’m somewhat argumentative”), anger (3 items; e.g., “I flare up quickly but get over it quickly”), and hostility (3 items; e.g., “I wonder why sometimes I feel so bitter about things”). Higher scores indicate higher levels of aggression. The Cronbach’s alpha values were as follows: physical aggression (α = 0.66), verbal aggression (α = 0.55), hostility (α = 0.72), and anger (α = 0.71). The Arabic version can be found in Appendix A Table A1.

**The Perseverative Thinking Questionnaire (PTQ).** The Perseverative Thinking Questionnaire [74] is composed of 15 items evaluating core features, mental resources, and unproductiveness due to repetitive negative thinking (e.g., “I think about many problems without solving any of them”, or “My thoughts repeat themselves”). Items were rated on a 5-point Likert scale ranging from 0 = “never” to 4 = “almost always”. A higher score on each dimension reflects a higher level of repetitive negative thinking. The Cronbach’s alpha values were as follows: core features (α = 0.92), mental resources (α = 0.83), and unproductiveness (α = 0.83). The Arabic version can be found in Appendix A Table A2.

### 2.5. Translation Procedure 

The forward and backward translation method was applied to different scales. The English version was translated into Arabic by a Lebanese translator who was unfamiliar with the questionnaire and the study. Then, a Lebanese psychologist with full working proficiency in English translated the Arabic version back into English. The initial and the second English versions were compared to detect and later eliminate any inconsistencies.

### 2.6. Statistical Analysis

Confirmatory factor analyses were conducted to test the four-factor and the three-factor structures of the BPAQ-SF and PTQ scales, respectively, that were found in the original validation studies. Confirmatory factor analysis was performed using RStudio (Version 1.4.1103 for Macintosh) and the Lavaan and semTools packages. We used the weighted least squares means and variance adjusted (WLSMV) estimation method, which is more appropriate for ordinal data. 

Data analysis was conducted using SPSS software version 23. No missing data was found, as all questions were required in the online survey. Cronbach’s alpha values were recorded for reliability analyses of all scales and subscales. All aggression subscale scores were normally distributed, with skewness and kurtosis values varying between −1 and +1 [100]. Student’s t and ANOVA tests were used to compare two, and three or more means, respectively. The Pearson correlation test was used to compare two continuous variables. To check for a significant indirect effect of PTQ between bullying victimization and aggression/hostility/anger, we conducted a path analysis using SPSS AMOS v.26. Variables that displayed a *p* < 0.25 in bivariate analysis were included in the path analysis. Significance was set at *p* < 0.05.

## 3. Results

### 3.1. Sociodemographic and Other Characteristics of Sample

Among 379 adolescent participants (mean age = 16.07 ± 1.19 years), 64.9% were females.

Other characteristics are summarized in Table 1.

### 3.2. Scale Validation

**Figure 1:**
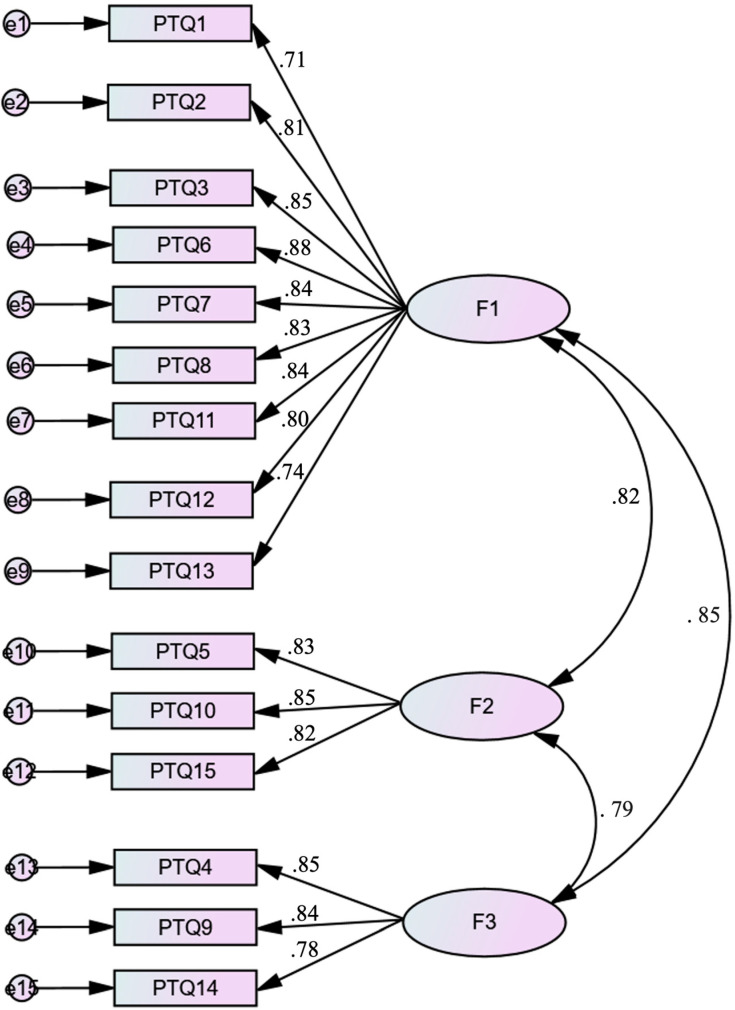
Standardized factor loadings of the three-factor model of the Arabic version of the Perseverative Thinking Questionnaire (PTQ) (*p* < 0.001 for all loading factors). F1 = PTQ core features, F2 = PTQ mental resources, F3 = unproductiveness.

The three-factor solution of the PTQ scale showed excellent model fit, with a CFI of 0.99 and a GFI of 0.99, an SRMR of 0.04, and an RMSEA of 0.08 (90% CI of RMSEA = 0.07, 0.09) (Figure 1). 

**Figure 2:**
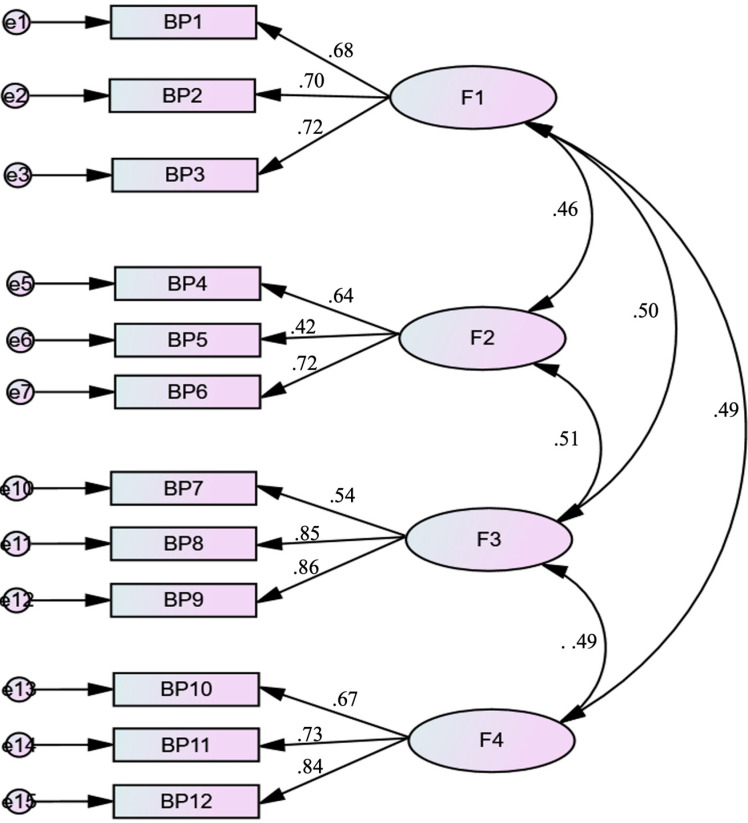
Standardized factor loadings of the four-factor model of the Arabic version of the Buss–Perry Aggression Questionnaire-Short Form (*p* < 0.001 for all loading factors). F1= Physical aggression, F2 = Verbal aggression, F3 = Anger, F4 = Hostility.

The four-factor solution of the BPAQ-SF also showed excellent model fit, with a significant CFI of 0.99 and a GFI of 0.99, an SRMR of 0.05, and an RMSEA of 0.06 (90% CI 0.05, 0.07) (Figure 2). 

### 3.3. Bivariate Analysis

The bivariate analysis results can be found in Table 2 and Table 3. In the current sample, males displayed greater mean physical aggression scores compared to females (7.03 vs. 6.36; *p* = 0.043), while females had higher mean anger scores compared to males (8.45 vs. 7.53; *p* = 0.009). Higher PTQ core features, mental resources, and unproductiveness were found to be significantly associated with more physical aggression, verbal aggression, anger, and hostility. Older age was significantly associated with more verbal aggression. Higher BMI was found to be significantly associated with more physical aggression, while more financial burden was significantly associated with greater hostility.

### 3.4. Path Analysis

**Figure 3:**
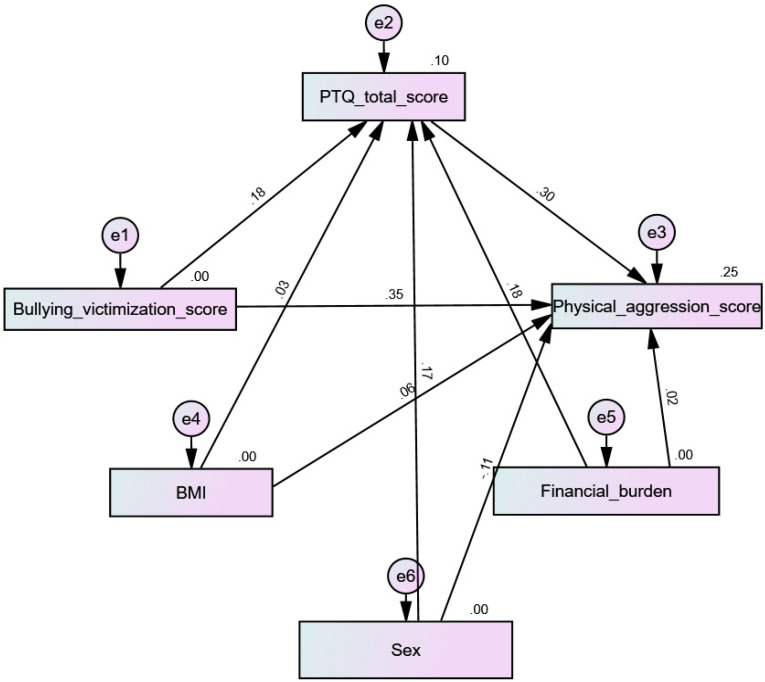
Path analysis model with physical aggression as the dependent variable.

**Figure 4:**
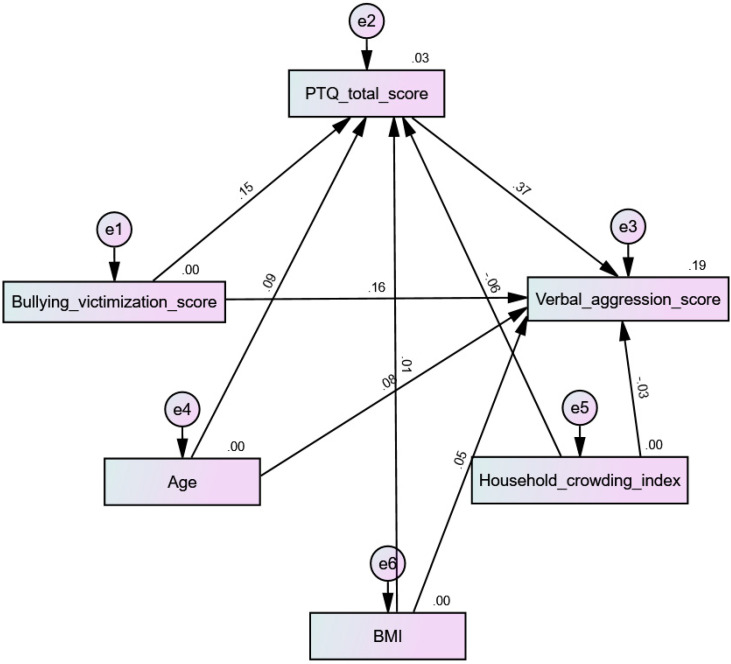
Path analysis model with verbal aggression as the dependent variable.

**Figure 5:**
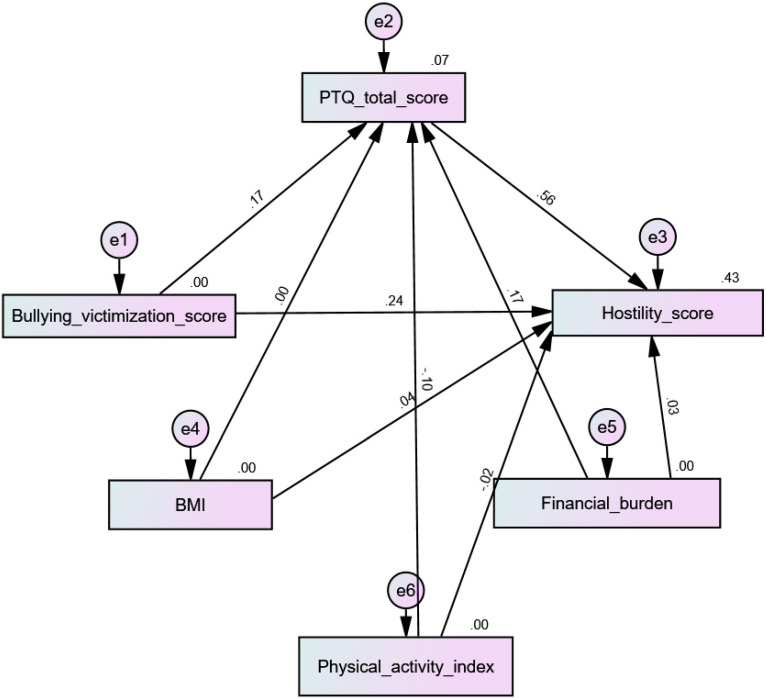
Path analysis model with hostility as the dependent variable.

**Figure 6:**
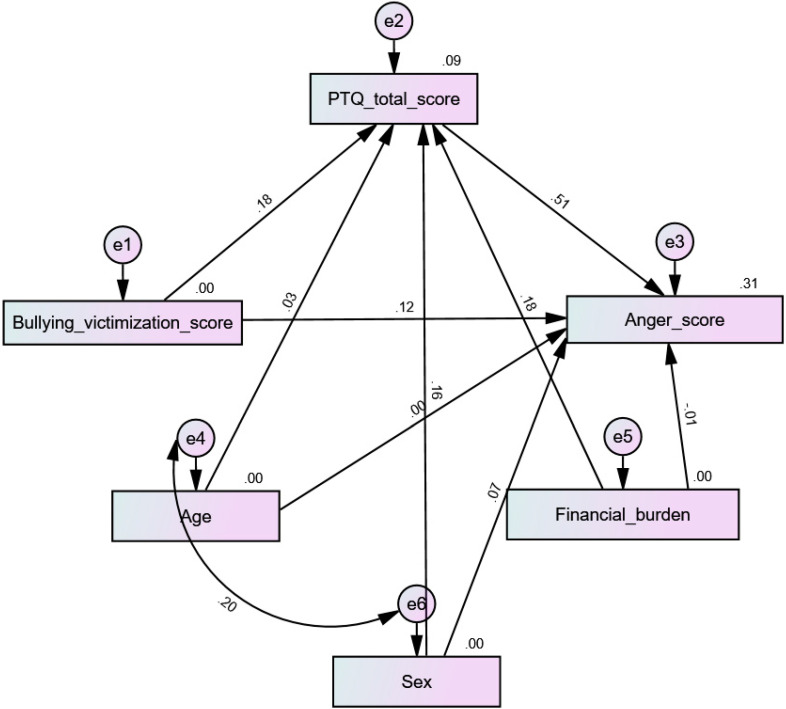
Path analysis model with anger as the dependent variable.

All four tested models had acceptable fit indices (Table 4). The results indicate that PTQ mediated the association between bullying victimization and physical aggression (beta = 0.053; 90% CI 0.03–0.09; *p* < 0.001) (Figure 3), verbal aggression (beta = 0.06; 90% CI 0.03–0.09; *p* = 0.001) (Figure 4), hostility (beta = 0.10; 90% CI 0.06–0.15; *p* = 0.001) (Figure 5), and anger (beta = 0.09; 90% CI 0.05–0.14; *p* = 0.001) (Figure 6).

## 4. Discussion

Bullying victimization has been recognized as a major and complex psycho-social problem [101] that requires considerable efforts from both public health professionals (practitioners, researchers, and educators) and the general public [102]. This paper represents an attempt to further understand the factors linked to aggressive tendencies among victimized adolescents. For this, one socio-cognitive factor, repetitive negative thinking, has been suggested as a potential mechanism explaining how victimization may be associated with aggressive behaviors among Lebanese adolescent students. As expected, we found that victimization not only directly contributed to students’ levels of aggression but also indirectly through repetitive negative thinking. 

This study also aimed to validate the PTQ and BPAQ-SF in Arabic. We found evidence that these scales have robust psychometric properties and are brief, easy-to-use assessment tools for Arab-speaking adolescents. The Arabic BPAQ-SF and PTQ revealed satisfactory internal consistencies, with a Cronbach’s alpha varying from 0.66 to 0.72 for the BPAQ-SF (except for the verbal aggression subscale, which revealed an α value of 0.55), and from 0.83 to 0.92 for the PTQ. In addition, the current results were consistent with those of the original versions’ validation studies, showing that the four-factor solution of the BPAQ-SF and the three-factor solution of the PTQ both revealed excellent model fit. Making these scales available to researchers from Arab countries would be beneficial to the whole research community, as ensuring the comparability of these assessment methods may help prevent wide variations in research findings related to these topics [85]. It would also encourage producing more research from the under-studied Arab world [103]. Our findings showed excellent model fit of the four-factor structure of the BPAQ-SF, unlike other previous validation studies that found poor fit (e.g., [81,104,105]). However, we found a poor but acceptable Cronbach’s alpha value [106] for the verbal aggression subscale. As previously said, aggression is a culturally dependent construct [92]; the low internal consistency of this subscale might be due to cultural variability and calls for further studies to extend its cross-cultural validity in other Arab contexts.

Regarding the direct effects, we found that higher IBS scores, translating into more frequent self-reported bullying victimization experiences by students during the past month, were significantly and positively correlated to more physical aggression, verbal aggression, anger, and hostility. These findings are in line with those from earlier cross-sectional and longitudinal studies showing that bullying victimization is positively associated with aggressive behavior among adolescents [35,43,44,107,108]. Different explanations have been advanced in literature to explain the positive link between being bullied and exhibiting aggressive behaviors. For example, it has been suggested that aggression serves as a buffer for negative emotional responses to bullying (e.g., anxiety and anger) [35,109]. It has also been suggested that isolation from peers caused by bullying victimization may lead to losing social skills, which leads in turn to externalizing problems [108]. Although the relationship between bullying victimization and aggression is well established, it is certain that not all victimized adolescents will evolve into aggressors [110]. However, a gap remains in factors and mechanisms that could help identify at-risk adolescents. To address this knowledge gap, we investigated the role of one potential mediator in this relationship, the repetitive negative thinking process.

In terms of mediation analyses, this study revealed that students who experienced more frequent victimization were more likely to display high levels of aggression themselves under the mediation of repetitive negative thinking. In other words, the overall indirect effect of bullying victimization on aggression through repetitive negative thinking (which included PTQ core features, mental resources, and unproductiveness as mediators) was significant. Similar to our findings, a recent longitudinal study performed in Finland in 2021 by Malamut and Salmivalli found that rumination about past victimization experiences mediated the positive prospective association between bully victimization and later bully perpetration [68]. There are several reasons to expect this pattern of findings. One reason is that adolescents who tend to repeatedly and negatively think about their victimization experiences are those most likely to have developed a vulnerability schema, which leads in turn to a desire to protect themselves against potential future victimization through aggression [111]. Indeed, the “victim schema model” suggests that peer victimization may result in biased cognitive and emotional regulation processes, which themselves lead to aggressive behavior as a response to perceived threat [112]. In sum, a few previous studies investigated the role of some socio-cognitive factors in pathways underlying the relationship between bullying victimization and subsequent aggression (e.g., [41,42,113]). However, no studies have examined so far the effects of repetitive negative thinking in this relationship. Also, all these studies focused on one specific type of aggression, which is bullying perpetration; and thus cannot be generalized to other forms of aggression. Therefore, our study is the first, to our knowledge, to document a mediating (indirect) effect of repetitive negative thinking on the link between bullying victimization and aggressiveness (in all its forms).

### 4.1. Study Implications

Interpersonal interactions have proven to play a determinant role in the development of children and adolescents [114]. Bullying victimization, a specific form of interpersonal violence, has devastating and long-lasting consequences for victimized students when occurring during early adolescence [115], including aggressive behaviors. Hence the importance of developing and implementing effective prevention interventions aiming to promote healthy relationships and positive interactions with peers, as well as decreasing bullying behaviors in schools. To date, antibullying policies have been inconsistent [116]; and school bullying prevention programs (such as the Olweus Bullying Prevention Program [117]) have produced mixed results in some countries (e.g., Germany, [118]), and have shown to be generally poorly effective among adolescents [53,54]. 

A first step towards developing new and effective interventions is understanding pathways leading from bullying victimization to aggression, which are still largely understudied and unknown. The present study sought to expand the literature on the role of socio-cognitive processes in the association between bullying victimization and aggression among adolescent students, by investigating the mediating effects of repetitive negative thinking. On the basis of the present findings, we could preliminarily confirm our hypothesis. Our results, along with prior data, suggest that routine and early detection of behavioral problems and other negative school experiences should be implemented in schools. 

When victimized students tend to repeatedly and negatively think about their adverse experiences, they would be most likely to turn to aggression. This suggests that therapies focused on negative thinking, such as repetitive negative thinking-focused ACT (Acceptance and Commitment Therapy Focused on Repetitive Negative Thinking) [119,120], may be promising avenues to prevent aggressive behaviors in students victimized by peers. This kind of therapy has been tested and shown to be effective in reducing emotional problems; however, to our knowledge, these approaches have not been previously analyzed for bullying problems. This calls for experimental studies to test our hypothesis. Finally, optimizing prevention and intervention programs and their implementation in schools requires that students, school staff, clinicians, researchers, and the whole community work together and coordinate their efforts [121]. 

### 4.2. Limitations and Strengths 

This study has certain limitations that can provide fruitful directions for future research. First, employing a cross-sectional design does not allow for establishing a causal relationship between victimization and aggression. Second, due to the self-report nature of the questionnaire used, reporting bias cannot be excluded. For greater validity, future studies might consider collecting bullying- and aggression-related information from peers, teachers, and parents. In addition, financial burden was assessed using a single question rather than a scale. Third, although we attempted to shed light on a previously unstudied mediator in the relationship between victimization and aggression, many other factors (such as drug use, family interactions, and school life satisfaction) might play key roles in this relationship and their mediating effects should be tested in future studies. Fourth, our findings showed an excellent model fit of the four-factor structure of the BPAQ-SF, unlike other previous validation studies that found a poor fit (e.g., [81,104,105]). However, we found a poor but acceptable Cronbach’s alpha [106] for the verbal and physical aggression subscales. As previously said, aggression is a culturally dependent construct [92], and the low internal consistency of this subscale might be due to cultural variability and calls for further studies to extend its cross-cultural validity in other Arab contexts.

This study has a number of strengths that deserve recognition. First, most of the previous studies examined bullying victimization in relation to bullying perpetration rather than aggression per se; while this study provides a broader overview of the topic by investigating victimization in relation to four aggression dimensions (i.e., physical aggression, verbal aggression, anger, and hostility). Second, by using valid measures of bullying victimization, aggression, and repetitive negative thinking that have been adapted to the local Lebanese context and culture, the present findings contribute reliable information to the field of bullying, allowing comparison with the findings of other studies locally, regionally, and internationally. Third, the study addressed an under-explored topic in the Arab population, especially the relationship between bullying victimization and aggression, given that each of these constructs have been researched independently in adolescent populations in previous Arab studies. 

## 5. Conclusions

Our study expands on past research by showing that repetitive negative thinking, a socio-cognitive factor that has proven to be impactful on students’ mental health, is one factor that underlies the cross-sectional relationship between bullying victimization and aggression. This suggests that interventions aiming at preventing aggressive behaviors among adolescents, in general, and students, in particular, may be more effective if focused on repetitive negative thinking. Compared to previous studies, we investigated four dimensions of aggression, not only bully perpetration, which might provide a clearer picture of the problem. The cross-sectional design remains, however, a major limitation, and calls for additional longitudinal studies to support our findings. Although we provided preliminary evidence suggesting repetitive negative thinking as a possible mediator of multiple forms of aggression after bullying victimization in adolescent students, future longitudinal research is needed to support our findings and deepen our understanding of the mechanisms underlying this relationship by investigating other pathways through which bullying victimization leads to aggression (e.g., other coping strategies). Furthermore, negative thinking subsequent to bullying victimization may also be associated with diverse other maladjustment outcomes, such as feelings of loneliness and social dissatisfaction, and impaired peer relationships and academic performance. Additional studies should consider the influence of bullying victimization and repetitive negative thinking on these outcomes.

## Figures and Tables

**Table 1:** Sociodemographic and other characteristics of the participants (*N* = 379).

Variable	N (%)
Sex	
Male	133 (35.1%)
Female	246 (64.9%)
	**Mean ± SD**
Age (in years)	16.07 ± 1.19
Physical activity index	27.78 ± 20.15
Household crowding index (persons/room)	1.26 ± 0.74
Body mass index (kg/m^2^)	22.33 ± 3.79
Financial burden	4.96 ± 2.80
Physical aggression	6.59 ± 2.90
Verbal aggression	7.46 ± 2.85
Anger	8.12 ± 3.29
Hostility	6.80 ± 3.12
PTQ core features	16.66 ± 8.86
PTQ mental resources	4.86 ± 3.25
PTQ unproductiveness	5.12 ± 3.27
Bullying victimization	3.30 ± 5.01

**Table 2:** Bivariate analysis of the categorical variables associated with aggression scores.

Variable	Physical Aggression	Verbal Aggression	Anger	Hostility
Sex				
Male	7.03 ± 3.29	7.50 ± 3.04	7.53 ± 3.22	6.55 ± 3.25
Female	6.36 ± 2.64	7.44 ± 2.76	8.45 ± 3.29	6.94 ± 3.04
*p*	**0.043**	0.842	**0.009**	0.251

Significant *p*-values are indicated in bold.

**Table 3:** Bivariate analysis of the continuous variables associated with the aggression scores.

Variable	Physical Aggression		Verbal Aggression		Anger		Hostility	
	r	*p*	r	*p*	r	*p*	r	*p*
Physical aggression	1	-						
Verbal aggression	0.46	**<0.001**	1	-				
Anger	0.50	**<0.001**	0.51	**<0.001**	1	-		
Hostility	0.49	**<0.001**	0.49	**<0.001**	0.67	**<0.001**	1	-
Bullying victimization	0.41	**<0.001**	0.23	**<0.001**	0.20	**<0.001**	0.33	**<0.001**
PTQ core features	0.35	**<0.001**	0.40	**<0.001**	0.54	**<0.001**	0.59	**<0.001**
PTQ mental resources	0.28	**<0.001**	0.34	**<0.001**	0.47	**<0.001**	0.56	**<0.001**
PTQ unproductiveness	0.30	**<0.001**	0.37	**<0.001**	0.48	**<0.001**	0.57	**<0.001**
Age	0.02	0.748	0.13	**0.014**	0.08	0.145	0.02	0.645
Physical activity index	0.03	0.547	−0.01	0.808	−0.04	0.442	−0.06	0.213
Household crowding index	−0.05	0.344	−0.07	0.172	0.04	0.475	−0.05	0.332
Body mass index	0.10	**0.043**	0.07	0.154	0.03	0.604	0.07	0.181
Financial burden	0.07	0.149	0.05	0.349	0.09	0.086	0.14	**0.006**

Significant *p*-values are indicated in bold; *r* = Pearson correlation coefficient.

**Table 4:** Fit indices of the path analyses.

Dependent Variable	χ^2^/df	*p*	CFI	SRMR	RMSEA	90% CI	Pclose
Physical aggression	23.81/6 = 3.97	<0.001	0.89	0.057	0.089	0.053–0.127	0.038
Verbal aggression	11.96/6 = 1.99	<0.001	0.94	0.039	0.051	0.001–0.094	0.421
Hostility	17.64/6 = 2.94	<0.001	0.95	0.047	0.072	0.034–0.112	0.151
Anger	15.78/5 = 3.15	<0.001	0.94	0.043	0.076	0.035–0.119	0.131

## Data Availability

The authors do not have the right to share any data information as per the ethics committee rules and regulations.

## Appendix A

**Table A1:** Translated items of the Buss-Perry Aggression Questionnaire- Short Form in Arabic language.

.باستخدام هذا المقياس المكوّن من 5 نقاط، وضّح كيف أن كلّ من العبارات التالية غير مميّزة أو مميّزة في وصفك. ضع تقييمك في المربّع المناسب					
يصفني للغاية	يصفني قليلاً	لا يمكن القول أنّه من سماتي ولكنّه أيضاً ليس من سماتي	لا يصفني قليلاً	يصفني للغاية	
					.قد أضرب شخصاً آخر إذا إستفزّني بما فيه الكفاية
					Given enough provocation, I may hit another person.
					.هناك أناس دفعوني كثيراً حتى وصلنا إلى التشاجر
					There are people who pushed me so far that we came to blows.
					.لقد هدّدت أناساً أعرفهم
					I have threatened people I know.
					.كثيراً ما أجد نفسي أختلف مع الناس
					I often find myself disagreeing with people.
					.لا أستطيع الدخول في جدال عندما يختلف الناس معي
					I can’t help getting into arguments when people disagree with me.
					.أصدقائي يقولون عنّي أنني إنسان مُجادل نوعاً ما
					My friends say that I’m somewhat argumentative.
					.أستشيط غضباً بسرعة لكنّني أتخطى ذلك بسرعة ايضاً
					I flare up quickly but get over it quickly.
					.في بعض الأحيان أفقد صوابي من دون أيّ سبب
					Sometimes I fly off the handle for no good reason.
					.أجد صعوبة في السيطرة على أعصابي
					I have trouble controlling my temper.
					.أحياناً أشعر كأنّي تلقّيت معاملة غير عادلة من الحياة
					At times I feel I have gotten a raw deal out of life.
					.يبدو أنّ الآخرين يحصلون دائمًا على فُرَص مؤاتية
					Other people always seem to get the breaks.
					.أتساءل لماذا أشعر أحيانًا بالمرارة تجاه الأشياء
					I wonder why sometimes I feel so bitter about things.

**Table A2:** Translated items of the Perseverative Thinking Questionnaire in Arabic language.

Almost Always تقريبا دائماً	Often غالباً	Sometimes بعض الأحيان	Rarely نادراً	Never أبداً	Perseverative Thinking Questionnaire (PTQ): Repetitive Negative Thinking
					The same thoughts keep going through my mind again and again.
					.نفس الأفكار تستمرّ في المرور في ذهني مرارًا وتكرارًا
					Thoughts intrude into my mind.
					.الأفكار تتطفلّ على ذهني
					I can’t stop dwelling on them.
					.لا أستطيع التوقّف عن الخوض /العيش في أفكاري
					I think about many problems without solving any of them.
					.أفكّر في العديد من المشاكل دون حلّ أي منها
					I can’t do anything else while thinking about my problems.
					.لا يمكنني فعل أي شيء آخر أثناء التفكير في مشاكلي
					My thoughts repeat themselves.
					.أفكاري تكرّر نفسها
					Thoughts come to my mind without me wanting them to.
					.الأفكار تتبادر إلى ذهني دون أن أرغب في ذلك
					I get stuck on certain issues and can’t move on.
					.أعلق في بعض القضايا ولا يمكنني المضي قدمًا
					I keep asking myself questions without finding an answer.
					.أستمرّ في طرح الأسئلة على نفسي دون أن أجد إجابة
					My thoughts prevent me from focusing on other things.
					.أفكاري تمنعني من التركيز على أشياء أخرى
					I keep thinking about the same issue all the time.
					.أبقى أفكّر في نفس المشكلة طوال الوقت
					Thoughts just pop into my mind.
					.تنبثق أو تظهر الأفكار فقط في عقلي
					I feel driven to continue dwelling on the same issue.
					.أشعر بالدافع لمواصلة الخوض/العيش في نفس القضية/المشكلة
					My thoughts are not much help to me.
					.أفكاري لا تساعدني كثيرا
					My thoughts take up all my attention.
					.أفكاري تستحوذ على كل انتباهي
